# Naturalistic stimuli in neuroimaging studies of autism spectrum disorder: a systematic review

**DOI:** 10.3389/fnhum.2026.1887914

**Published:** 2026-07-03

**Authors:** Ioannis Ntoumanis, Christos Papadelis

**Affiliations:** 1 Jane and John Justin Institute for Mind Health, Cook Children’s Health Care System, Fort Worth, TX, United States; 2 Department of Bioengineering, University of Texas at Arlington, Arlington, TX, United States; 3 Department of Pediatrics, Burnett School of Medicine, Texas Christian University, Fort Worth, TX, United States

**Keywords:** autism spectrum disorder, functional connectivity, intersubject correlation, naturalistic stimuli, neuroimaging, social cognition

## Abstract

Naturalistic stimuli, such as movies and socially rich videos, are increasingly used in neuroimaging to study brain function under conditions that approximate real-world experience. This approach may be particularly informative for autism spectrum disorder (ASD), where social and emotional differences often emerge in complex, dynamic environments that simplified paradigms may not adequately capture. Here, we provide a systematic review of neuroimaging studies that have employed naturalistic stimuli in ASD research. Our goals are to: (i) summarize existing findings; (ii) identify convergent neural findings across different data analysis methods; and (iii) clarify conceptual and methodological limitations that constrain interpretation. Across studies, autistic individuals often show reduced intersubject neural synchronization during naturalistic viewing, particularly in brain regions supporting social cognition and theory of mind. Multiple reports additionally indicate alterations in large-scale functional network organization, although results vary with stimulus type and data analysis used. Some studies have also linked these neural differences to behavioral measures, including autistic traits. Together, these findings suggest that naturalistic stimuli offer a promising framework for capturing heterogeneity in ASD while also introducing challenges related to experimental design and interpretation. We conclude by outlining methodological recommendations to guide future work in this developing field.

## 1 Introduction

Autism spectrum disorder (ASD) is a neurodevelopmental condition characterized by persistent difficulties in social communication and interaction, alongside restricted and repetitive patterns of behavior (American Psychiatric Association, 2013; Lord et al., 2020). These difficulties are not static or context-independent, but they rather emerge most clearly in dynamic environments that require the continuous integration of social, affective, and contextual signals over time (Pelphrey et al., 2011; Volkmar, 2011). Understanding the neural mechanisms underlying these difficulties has therefore been a central goal of cognitive neuroscience. However, the majority of neuroimaging studies examining social and emotional processing in ASD have relied on artificial, decontextualized stimuli, most commonly static pictures of facial expressions presented in isolation (Tang et al., 2020; McPartland et al., 2021). Although these approaches have substantially advanced our understanding of the autistic brain, they isolate social cues from the situations in which they normally occur, and may therefore provide an incomplete picture of how autistic individuals process social information in real-world settings.

Despite extensive work using task-based and resting-state paradigms, findings in ASD neuroimaging remain heterogeneous, particularly regarding large-scale functional connectivity and its relevance to behavior (Uddin et al., 2013; Di Martino et al., 2014; Hull et al., 2017). Resting-state studies have implicated altered default mode, salience, and social brain networks in ASD, yet the direction and consistency of these effects vary across samples and analytical choices (Nomi and Uddin, 2015; Hull et al., 2017). Similarly, task-based studies have identified differences in brain areas supporting face perception, theory of mind, and emotion processing, but these effects are often subtle and vary depending on task and stimulus characteristics (Philip et al., 2012; Redcay and Schilbach, 2019). This variability suggests that artificial, decontextualized stimuli may fail to capture the contextual complexity in which social and emotional difficulties in ASD manifest, pointing to the need for more ecologically valid experimental approaches.

Naturalistic stimuli, such as movies or socially rich videos, provide an alternative framework by preserving the temporal and contextual complexity of real environments while maintaining experimental control (Hasson and Honey, 2012; Sonkusare et al., 2019). Unlike conventional paradigms using decontextualized stimuli, which average neural responses across brief, isolated trials, naturalistic stimuli engage multiple perceptual, cognitive, and affective systems simultaneously, enabling researchers to examine how neural responses evolve continuously over time (Nastase et al., 2019; Meer et al., 2020; Jääskeläinen et al., 2021). This temporal dimension is important for studying social cognition, which depends on the integration of information across extended periods rather than responses to discrete events (McMahon and Isik, 2023). Passive naturalistic viewing also removes the confound of explicit task instructions, which can constrain neural responses and reduce ecological validity (Finn et al., 2020). Compared with resting-state paradigms, naturalistic stimuli offer an additional advantage. They provide a shared external drive that grounds cross-individual and cross-group comparisons in a common experience, while simultaneously engaging the social, emotional, and mentalizing systems most relevant to understanding real-world behavior. Unlike rest, neural responses during naturalistic viewing can be linked to specific stimulus features, such as the presence of faces, emotional intensity, or speech content, allowing researchers to identify not only whether neural responses differ between groups, but when and under what conditions those differences emerge (Bolton et al., 2020; Nummenmaa et al., 2012). Movie-watching has also been shown to outperform resting-state paradigms for functional connectivity-based prediction of individual behavioral differences (Finn and Bandettini, 2021). Recent theoretical and empirical work has formalized these principles into a framework for naturalistic neuroimaging, establishing that continuous shared stimuli enable quantification of how similarly different individuals process the same unfolding experience, and that individual differences in these shared neural responses reflect meaningful variation in cognition and behavior (Nastase et al., 2019; Jääskeläinen et al., 2021, 2022; Saarimäki, 2021).

These properties make naturalistic stimuli particularly promising for the study of ASD. Because the social and emotional difficulties that characterize autism are inherently context-dependent, they may be probed most effectively with stimuli that preserve the dynamic context in which they arise rather than stripping it away (Pelphrey et al., 2011; Volkmar, 2011). ASD is also a highly heterogeneous condition, and ISC-based analyses, which quantify the degree to which individuals respond similarly to a shared stimulus, are well-suited to capturing this variability in a data-driven way, without imposing *a priori* assumptions about which stimulus features drive neural differences (Nastase et al., 2019; Finn et al., 2020). Examining neural responses to naturalistic stimuli may therefore provide insights into dynamic social information processing that are difficult to obtain with either conventional or resting-state designs. Despite growing interest in naturalistic neuroimaging more broadly, applications to ASD remain relatively sparse and methodologically diverse. Moreover, findings across these studies have not been systematically compared, making it difficult to determine which neural findings are consistent, which depend on analysis methods or design choices, and whether naturalistic stimuli provide novel insights relative to conventional approaches.

Although interactive and second-person paradigms, in which participants engage in live social exchanges, may capture the neural underpinnings of social and emotional processing with even greater ecological validity than passive movie watching (Czekóová et al., 2025), they introduce participant-dependent contingencies that preclude exact stimulus replication across individuals, and they are considerably more technically demanding. The present review therefore focuses specifically on prerecorded naturalistic stimuli, consistent with prior reviews in this area (Jääskeläinen et al., 2021, 2022; Saarimäki, 2021).

The aim of this review is therefore threefold: (i) to provide a comprehensive and up-to-date overview of all available neuroimaging studies employing naturalistic stimuli in ASD; (ii) to identify the most consistent neural findings across studies; and (iii) to evaluate methodological limitations that need to be addressed in future studies. Finally, this review provides a structured framework for interpreting and designing future naturalistic neuroimaging studies in ASD.

## 2 Methods

This systematic review was designed and reported in accordance with the Preferred Reporting Items for Systematic reviews and Meta-Analyses (PRISMA) guidelines (Liberati et al., 2009).

### 2.1 Search strategy

Studies were identified by searching electronic databases and screening reference lists. Web of Science, Scopus, PubMed, and Google Scholar were queried using the following Boolean search terms:

(autism) AND (brain OR neural) AND (movie* OR film* OR “audio narrative”*)

These search terms were derived from seminal work on naturalistic neuroimaging in autism. No restrictions were placed on year of publication or article type. Reference lists of all eligible articles and reviews were additionally screened to identify further studies. The last search was conducted on March 2, 2026.

### 2.2 Eligibility criteria

Studies were included if they:

- Were published in a peer-reviewed journal;
- Used dynamic audiovisual, visual, or auditory stimuli featuring human actors (e.g., films, TV episodes, video clips, audio narratives);
- Included participants diagnosed with ASD alongside a non-autistic comparison group.

Studies were excluded if they:

- Used non-human or abstract stimuli (e.g., geometric animations or “inscapes”). The reason for this exclusion was that such stimuli may not reliably engage the social and affective processes central to ASD (Vanderwal et al., 2015; Redcay and Schilbach, 2019);
- Focused exclusively on live or interactive social exchanges rather than prerecorded naturalistic stimuli. The reason for this exclusion was that such paradigms introduce reciprocal behavior, variable task demands, and participant-dependent contingencies that fundamentally alter neural dynamics and reduce cross-study comparability with passive movie-viewing designs. In line with this rationale, prior reviews on naturalistic neuroimaging have also excluded interactive paradigms (Jääskeläinen et al., 2021, 2022; Saarimäki, 2021);
- Were case studies, letters to the editor, or conference abstracts.

**Figure 1:**
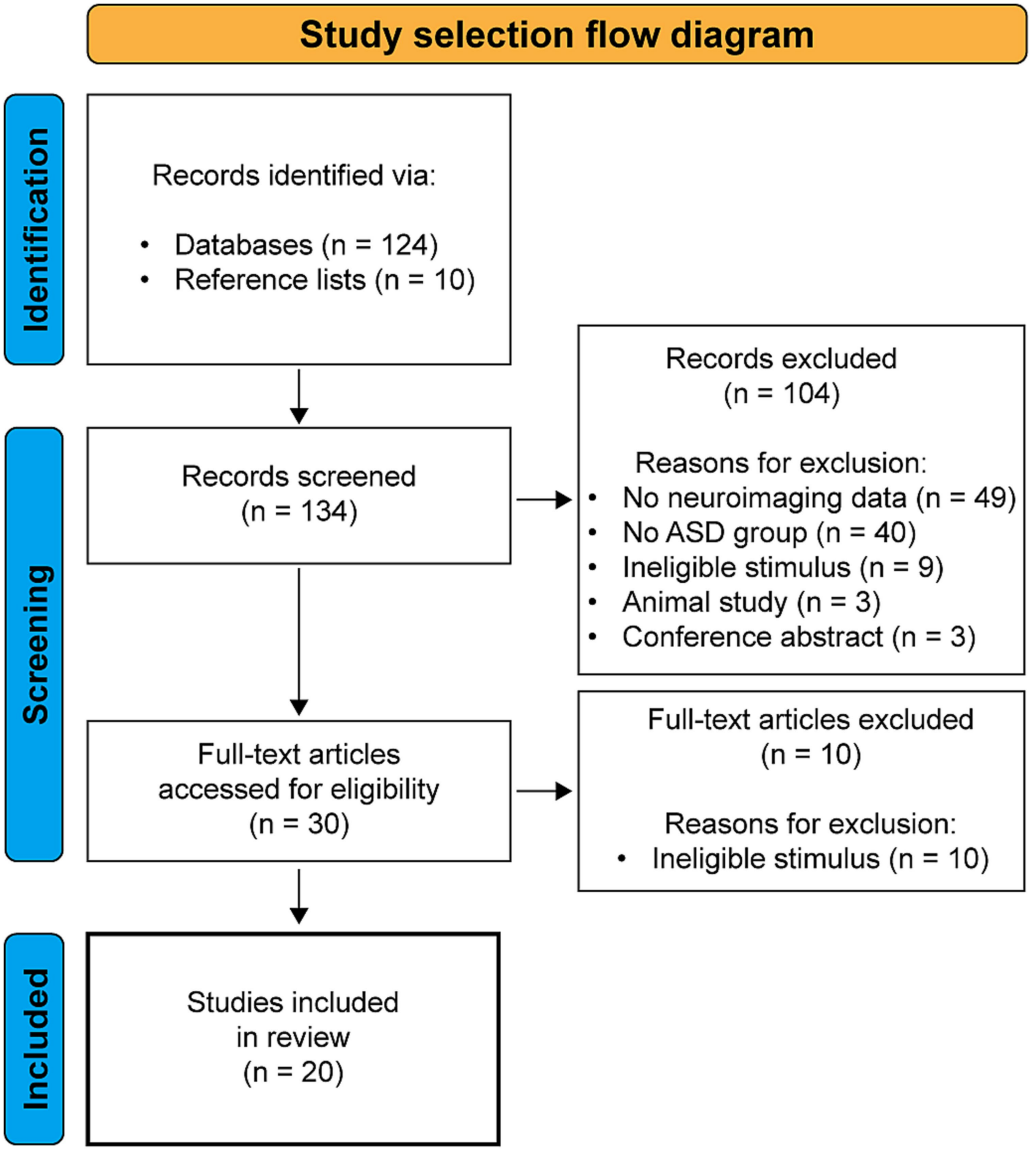
PRISMA flow diagram of study selection for systematic review of published research on naturalistic neuroimaging in autism spectrum disorder.

Titles and abstracts were first screened against the criteria. Full texts of potentially eligible articles were then reviewed, and additional exclusions were applied as necessary. The study selection process is summarized in Figure 1. Titles and abstracts were first screened against the eligibility criteria by two independent reviewers (I.N. and C.P.). Full texts of potentially eligible articles were then independently evaluated by both reviewers. The two reviewers reached full agreement on the final set of included studies.

Notable studies that were identified during screening but excluded include Vandewouw et al. (2021), which used inscapes, an abstract non-social animation designed to reduce head motion rather than to engage social or affective processing, and Camacho et al. (2024), which focused on social anxiety without directly comparing autistic and non-autistic groups. Although the term “naturalistic stimuli” broadly encompasses live and interactive paradigms, the present review uses it specifically to refer to prerecorded audiovisual, visual, and auditory dynamic stimuli, consistent with prior reviews in this area (Jääskeläinen et al., 2021, 2022; Saarimäki, 2021).

### 2.3 Data extraction

Data were extracted from each included study by I.N. and verified by C.P. For each study, the following information was recorded: neuroimaging modality, participant age group, stimulus type, stimulus duration, stimulus modality, data analysis methods, and main findings including brain regions involved (Table 1). No additional data were sought from study investigators.

**Table 1:** Summary of neuroimaging studies using naturalistic stimuli in individuals with autism spectrum disorder (ASD).

Study	Neuroimaging method	Participants’ age group	Stimulus type; Duration; Modality	Data analysis methods	Main findings; brain areas involved
Hasson et al. (2009)	fMRI	Adults	Movie clip; 10 min; Audiovisual	ISC; IaSC	Reduced ISC in ASD; V1, A1, LOC, ITS, PA, FFA, Precuneus, TOS, STS
Scherf et al. (2010)	fMRI	Adolescents	Movie clips; 15 s; Visual-only	GLM	Reduced activity in ASD; FFA, Fusiform
Salmi et al. (2013)	fMRI	Adults	Full movie; 67 min; Audiovisual	ISC	Reduced ISC in ASD; SMG, LOC, CN, PCC, Precuneus, ACC, SMA, NA, Insula
Byrge et al. (2015)	fMRI	Adults	Movie clip; 22 min; Audiovisual	ISC	Reduced ISC in ASD, further associated with social comprehension; PFC, PCC, TPJ, and other DMN regions
Pantelis et al. (2015)	fMRI	Adults	Movie clip; 21 min; Audiovisual	GLM; FC	Reduced activity and FC in ASD; TPJ, STS, OFC, TP
Glerean et al. (2016)	fMRI	Adults	Full movie; 68 min; Audiovisual	FC	Reorganization of subnetworks in ASD, further associated with autism spectrum measures; VTL, DMN, AUD, DA, V1
Rosenblau et al. (2016)	fMRI	Adults	Movie clips; 9–31 s; Audiovisual	GLM	Reduced activity in ASD; Amygdala
Bolton et al. (2018)	fMRI	Adolescents and adults	Movie clips; 300–353 s; Audiovisual	ISFC	Reduced ISFC in ASD; STS, MTG, ITG, occipital
Bolton et al. (2020)	fMRI	Adolescents and adults	Movie clips; 300–353 s; Audiovisual	ISFC	ASD participants show heterogeneous network recruitment, and ISFC relates to autism spectrum measures.
Lyons et al. (2020)	fMRI	Children and adolescents	Movie clip; 10 min; Audiovisual	ISC	Reduced ISC in ASD, further associated with autistic traits; TPJ, Precuneus, STS, PFC, Hippocampus
Ramot et al. (2020)	fMRI	Adolescents and adults	Movie clips; 14 s; Audiovisual	ISC	Reduced ISC in ASD; TPJ, MTG, STS, IFG, ACC, PCC
Kotila et al. (2020)	fMRI	Adults	Movie clips; 50–84 s; Audiovisual	ICA	Altered responses to multimodal cues in ASD; Insula, ACC
Ou et al. (2022)	fMRI	Children, adolescents, and adults	Movie clip; 10 min; Audiovisual	ISC; ISFC	Reduced ISC and ISFC in ASD for some segments of the movie, but increased for others; MTG, cerebellum, precuneus, CN, TP
Esfahlani et al. (2022)	fMRI	Adolescents and adults	Movie clips; 1–5 min; Audiovisual	Dynamic FC	Reduced dynamic FC bursts in ASD
Matsuzaki et al. (2022)	MEG	Children and adolescents	Movie clips; 10 s each; Audiovisual, Visual-only	Activated intensities; TFA	Altered activity in ASD, further associated with sensory profiles; STS, occipital, temporal
Ross et al. (2024)	fMRI	Children, adolescents, and adults	Movie clips; 8–22 s; Audiovisual, Audio-only, Visual-only	GLM	Increased activity in ASD; PFC, PCC, ACC
Turner et al. (2025)	fMRI	Adults	Movie clip; 5 min; Audiovisual	ISC; FC	Reduced FC and ISC in ASD, but not associated with behavioral performance; ToM
Mangnus et al. (2025)	fMRI	Adults	Movie clip; 6 min; Audiovisual	ISC	Increased ISC in ASD; SMG, ITG, CG
Zimmer et al. (2025)	fMRI	Adults	Movie clip; 6 min; Visual-only	IaSC	Lower repetition suppression in ASD for one scene; ToM
Lin et al. (2026)	fMRI	Adults	Movie clips; 9–22 s; Audiovisual, Visual-only	ISFC	Altered ISFC in ASD; Lower in visual, parietal, and frontal regions; higher in temporal regions

A1, primary auditory cortex; ACC, anterior cingulate cortex; AUD, auditory network; CG, calcarine gyrus; CN, caudate nucleus; DMN, default mode network; FC, functional connectivity; FFA, fusiform face area; GLM, general linear model; IaSC, intrasubject correlation; ICA, independent component analysis; ISC, intersubject correlation; ISFC, intersubject functional connectivity; ITS, inferior temporal sulcus; LOC, lateral occipital cortex; MTG, middle temporal gyrus; NA, nucleus accumbens; OFC, orbitofrontal cortex; PA, parahippocampal area; PCC, posterior cingulate cortex; SMA, supplementary motor area; SMG, supramarginal gyrus; STS, superior temporal sulcus; TFA, time frequency analysis; ToM, theory of mind network; TOS, transverse occipital sulcus; TP, temporal pole; V1, primary visual cortex; VTL, ventro-temporal limbic network. Age groups were categorized as “children” if participants included individuals younger than 10 years, “adolescents” if participants included individuals between 10 and 18 years, and “adults” if participants included individuals older than 18 years. When a study included more than one age group, all relevant categories are listed.

### 2.4 Risk of bias assessment

A risk-of-bias assessment was performed using the JBI Critical Appraisal Checklist for analytical cross-sectional studies (Moola et al., 2020). Each of the twenty included studies was assessed by I.N. and verified by C.P.

## 3 Results

### 3.1 Study characteristics

A total of twenty studies were included in this review (Table 1). Overall, the methodological quality of the included studies met the JBI standards (Supplementary Table S1). The most common strengths were valid outcome measurement and appropriate statistical analysis. The most frequent limitations were insufficient reporting of diagnostic instruments used to confirm ASD and inadequate management of confounders, particularly IQ differences between groups.

Geographically, the included studies spanned eight countries across North America, Europe, and East Asia, with the largest contributions from the United States (*n* = 8) and Finland (*n* = 4), followed by Germany (*n* = 3), Switzerland (*n* = 2), and one study each from Canada, China, Japan, and the Netherlands (Supplementary Figure S1). This geographic diversity highlights the growing international interest in applying naturalistic stimuli to autism research.

Most studies used functional magnetic resonance imaging (fMRI) with movie-based stimuli, whereas one study used magnetoencephalography (MEG). Across these studies, various data analysis methods were used, including intersubject correlation (ISC), intersubject functional connectivity (ISFC), dynamic functional connectivity, independent component analysis (ICA), general linear modeling (GLM), and time frequency analysis (TFA). ISC quantifies the similarity of neural responses across individuals viewing the same stimulus. ISFC extends this by correlating activity in one brain region with activity in other brain regions averaged across participants, isolating stimulus-driven connectivity. Dynamic functional connectivity characterizes how inter-regional coupling changes over time. ICA decomposes signals into spatially or temporally independent components. GLM tests for activation associated with specific events or features within the stimulus. TFA characterizes how oscillatory power changes across frequency bands over time. The sections below describe these analytical approaches in more detail and synthesize the main findings according to each.

### 3.2 Reduced intersubject correlation in autism

**Figure 2:**
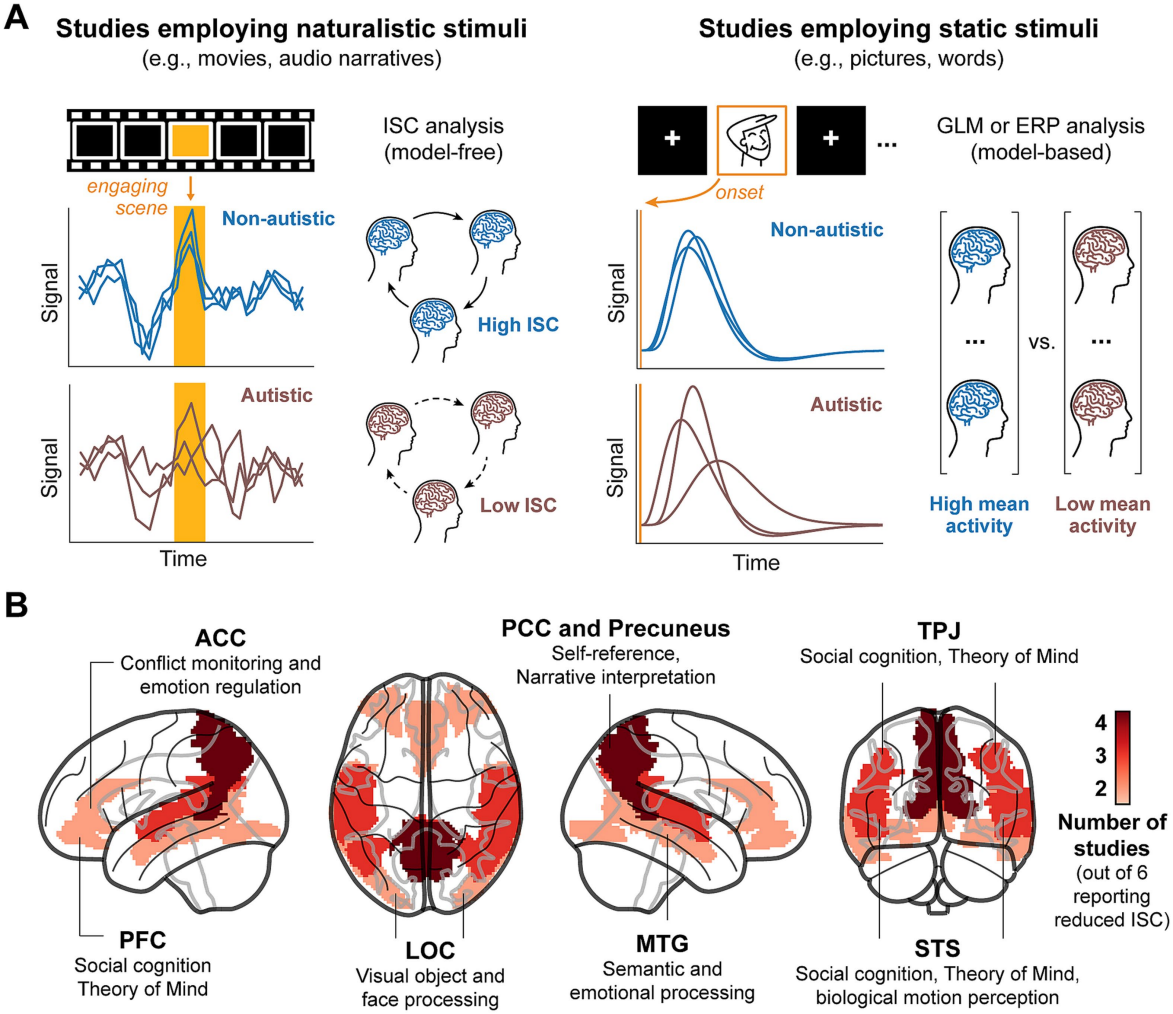
Conceptual and empirical overview of naturalistic neuroimaging findings in autism spectrum disorder (ASD). **(A)** Conceptual illustration of the methodological differences between paradigms employing naturalistic or static stimuli in autism research. Studies employing naturalistic stimuli (left) use continuous, socially rich stimuli, such as movies, capturing neural synchronization across participants. These designs typically reveal higher intersubject correlation (ISC) among non-autistic individuals and lower among individuals with ASD. Conventional paradigms (right) rely on simplified designs and use within-group averaging, which reduces sensitivity to individual variability. GLM, General Linear Modeling; ERP, event-related potential. **(B)** Brain regions where individuals with ASD frequently show reduced ISC. Regions were defined using the AAL atlas, and were included based on anatomical labels reported in the text of each study (e.g., ‘superior temporal sulcus’, ‘temporoparietal junction’) rather than on stereotaxic coordinates. Only ROIs mentioned in at least two studies were included. Six studies reporting reduced ISC contributed to this map. One study (Mangnus et al., 2025) reporting increased ISC in ASD is discussed in the text but not shown here. Warmer colors indicate a higher number of studies reporting each region (Precuneus = 5; Superior Temporal Sulcus (STS) = 5; Temporoparietal Junction (TPJ) = 4; Posterior Cingulate Cortex (PCC) = 3; Lateral Occipital Cortex (LOC) = 3; Anterior Cingulate Cortex (ACC) = 3; Prefrontal Cortex (PFC) = 2; Middle Temporal Gyrus (MTG) = 2; Fusiform Gyrus = 2). Functionally, these regions mainly support social cognition, Theory of Mind (ToM), emotional, and self-referential processing.

One of the most widely used data analysis methods in naturalistic neuroimaging is ISC (Hasson et al., 2004). This is a data-driven approach that quantifies the similarity of neural activity across different subjects exposed to the same continuous stimulus (Nastase et al., 2019). High ISC values indicate a highly consistent response across subjects, whereas low ISC values reflect a more idiosyncratic pattern of brain activity (Figure 2A). However, the interpretation of ISC depends critically on how it is computed, and two distinct implementations have been used in autism research. In the first, a cross-group ISC approach, the neural timeseries of each autistic participant is correlated with the average timeseries of non-autistic participants. Low cross-group ISC in this case indicates that autistic individuals respond differently from controls, but says nothing about whether autistic individuals are consistent with each other. In the second, a within-group ISC approach, each participant’s timeseries is correlated with the mean of all other members of the same group. Low within-group ISC in the autistic sample specifically indicates idiosyncratic, heterogeneous responses among autistic individuals. These two approaches address fundamentally different questions and should not be conflated. Across studies, reduced ISC in ASD has frequently been reported, particularly in brain regions associated with social cognition and higher-order processing (Hasson et al., 2009; Salmi et al., 2013; Byrge et al., 2015; Lyons et al., 2020; Ramot et al., 2020; Ou et al., 2022). Most of these studies computed cross-group ISC, comparing the neural timeseries of each autistic participant against the non-autistic group average. Early work by Hasson et al. (2009), which reported both cross-group and within-group ISC, showed lower cross-group ISC in visual and social perceptual areas in autistic adults. Salmi et al. (2013), also reporting both measures, observed more widespread reductions across temporal, parietal, and default mode regions during extended movie viewing. Byrge et al. (2015) extended these findings by showing that lower cross-group ISC in these areas was associated with poorer social comprehension, linking neural variability to behavioral differences.

Similar patterns have been reported across developmental samples. Reduced ISC has been reported in frontoparietal and theory of mind (ToM) networks in autistic children (Lyons et al., 2020), with the effects being more pronounced in a subgroup of participants with more pronounced autistic traits. Studies involving mixed-age cohorts spanning childhood to adulthood reported similar findings (Ramot et al., 2020; Ou et al., 2022). Although based on a small number of studies, these findings suggest that altered neural synchronization relative to non-autistic peers may not be limited to a specific developmental stage but may characterize ASD across the lifespan.

A schematic illustration of the brain regions most frequently reported to show reduced ISC in autism is shown in Figure 2B using the Automated Anatomical Labeling atlas (AALv3; Rolls et al., 2020). Functionally, these regions mainly support social cognition, theory of mind, emotional, and self-referential processing (Northoff et al., 2006; Schurz et al., 2014; Ntoumanis et al., 2026). This visualization is intended only to illustrate areas of convergence across studies. Regions were identified based on the anatomical labels reported in each study’s text and mapped to the nearest corresponding AAL parcel (no stereotaxic coordinates were used). Exact spatial locations and statistical thresholds may vary across experiments and, therefore, should not be interpreted as precise anatomical correspondence.

Importantly, results are not uniform. For example, Mangnus et al. (2025) reported that participants with ASD showed higher within-group ISC compared to participants without ASD in the supramarginal gyrus and inferior temporal gyrus. Similarly, Turner et al. (2025), who reported both cross-group and within-group ISC, found reduced ISC within the theory of mind network but noted that this reduction was not significantly associated with behavioral performance on social inference tasks. Segment-specific increases or decreases in ISC have been reported, particularly during socially intense or emotionally salient scenes that may require mental state inference (Ou et al., 2022; Zimmer et al., 2025). Thus, synchronization patterns depend on both the ISC approach used and stimulus content, and caution is needed against interpreting reduced ISC as a universal property of ASD.

In addition to ISC, several studies have examined intrasubject correlation (IaSC), which measures the consistency of neural responses within the same individual across repeated viewings (Hasson et al., 2004). This metric captures the reliability of stimulus-driven responses independent of alignment across participants. Hasson et al. (2009) showed that although autistic individuals exhibited lower cross-group ISC relative to non-autistic individuals, their IaSC was high, meaning that each autistic individual’s neural responses were consistent across repeated viewings of the same stimulus. One speculative interpretation is that this pattern could relate to the repetitive behavior and processing often described in ASD (Tian et al., 2022; Cooper et al., 2022), though this remains to be empirically tested. A similar pattern was observed by Zimmer et al. (2025) for most of the film, except for a single movie scene. This dissociation between preserved IaSC and reduced cross-group ISC suggests that the neural responses of autistic individuals may be consistently organized differently rather than simply noise. Experimental designs employing naturalistic stimuli are particularly well-suited to reveal this combination because they provide continuous stimulation, allowing neural similarity to be measured both across individuals and across repeated exposures.

When interpreted in the context of the broader autism neuroimaging literature, these ISC findings largely implicate regions that have also been identified by conventional studies using decontextualized stimuli. Meta-analyses of social cognition tasks consistently report atypical activation in anterior cingulate, medial prefrontal, insular, temporoparietal, and fusiform regions in ASD (Di Martino et al., 2014; Patriquin et al., 2016; Chen et al., 2024), many of which overlap with the areas showing reduced ISC during naturalistic viewing (Figure 2B). However, studies using naturalistic stimuli offer more than confirmation of previously implicated regions. They allow researchers to determine when neural divergence emerges during socially complex stimulation and whether that divergence is sustained or context-dependent. This emphasis on temporal dynamics is important because social understanding requires integration of information across scenes (McMahon and Isik, 2023; Martynenko et al., 2025). Conventional task-based contrasts typically average responses across brief trials and therefore cannot capture how group differences fluctuate across narrative segments (Figure 2A).

Overall, current evidence suggests that neural responses during naturalistic stimulation are often less synchronized across autistic individuals while remaining relatively reliable within individuals. This pattern aligns with behavioral evidence of heterogeneity in social perception and cognition across the autism spectrum (Chevallier et al., 2012; Hernandez et al., 2015; Kim et al., 2024). Importantly, the number of available studies remains modest, and designs vary considerably, which limits strong conclusions and underscores the need for replication and methodological standardization.

### 3.3 Brain network reorganization in autism

Modern views of cognition suggest that it depends not only on local activity patterns but also on how strongly key brain regions communicate with each other (Bressler and Menon, 2010; Cooper et al., 2017; Ntoumanis et al., 2024). In autism research, functional connectivity approaches have therefore played a central role in examining large-scale neural organization (Di Martino et al., 2014; Hull et al., 2017; Benkarim et al., 2021). While much of this work has relied on resting-state data, neuroimaging studies employing naturalistic stimuli are increasingly being used to investigate whether connectivity is altered in ASD during ecologically valid experiences (e.g., Bolton et al., 2018; Ou et al., 2022).

Several studies have applied conventional within-subject functional connectivity analyses to neuroimaging data acquired during naturalistic movie watching using interregional correlations and edge-centric metrics (e.g., Glerean et al., 2016; Esfahlani et al., 2022). For example, Pantelis et al. (2015) computed interregional correlations and reported reduced functional connectivity between temporoparietal and orbitofrontal regions in ASD during socially rich movie viewing. Similarly, Glerean et al. (2016) used correlation-based network analysis and observed atypical subnetwork organization in ASD, including increased connectivity within sensory and limbic systems and altered integration of socioemotional networks. Extending this work, Esfahlani et al. (2022) applied an edge-centric approach to examine time-resolved connectivity patterns and reported fewer transient high-amplitude connectivity events in ASD, indicating reduced moment-to-moment coordination across networks. Finally, Turner et al. (2025) used graph theory to characterize large-scale network organization and found reduced connectivity within theory of mind regions during movie viewing, an effect that was not significantly associated with behavioral performance on social inference tasks. These findings suggest that connectivity differences did not reflect a simple global reduction, but rather alterations that are network-specific and often depend on stimulus content.

The results summarized above come from studies that used within-subject functional connectivity, which can be influenced by participant-specific intrinsic dynamics, head motion, and physiological noise, making it difficult to isolate stimulus-driven interactions (Simony et al., 2016). This limitation can be addressed by a related but analytically distinct approach called intersubject functional connectivity (ISFC). ISFC correlates activity in one region of a participant with activity in another region averaged across a reference group of other participants (Simony et al., 2016; Nastase et al., 2019). As with ISC, it is important to note which participants constitute the reference group. Some studies use the non-autistic group as the reference to quantify how much each autistic participant’s inter-regional coupling aligns with a neurotypical template, while others use a leave-one-out approach within each group to quantify group-typical patterns separately. Using ISFC, Bolton et al. (2018, 2020) and Ou et al. (2022) reported reduced connectivity in ASD, particularly within the default mode network (DMN) and regions implicated in social cognition, such as the temporoparietal junction (TPJ) and prefrontal cortex (PFC), using non-autistic participants as the reference group. This suggests that stimulus-driven communication between brain areas, which typically underlies social and emotional processing (Saarimäki, 2021), is less aligned with the neurotypical template in autistic individuals during movie viewing. Bolton et al. (2020) further incorporated scene-level annotations of the movie stimuli and demonstrated that ISFC states covary with the presence of faces on the screen, among other stimulus features. In a cross-national study, Lin et al. (2026) demonstrated that both German and Finnish adults with ASD exhibited reduced ISFC relative to the non-autistic reference group in visual, parietal, and frontal regions when viewing short movie clips. However, ISFC in temporal regions was higher in the ASD group when movies were presented with sound, which the authors attributed to speech processing (Lin et al., 2026).

Developmentally, network reorganization in ASD has not been reported only in adults (Pantelis et al., 2015; Glerean et al., 2016; Turner et al., 2025), but also in mixed-age cohorts spanning childhood to adulthood (Bolton et al., 2018, 2020; Esfahlani et al., 2022; Ou et al., 2022). Although purely pediatric samples remain limited, current evidence suggests that altered stimulus-driven network reorganization is observable across developmental stages.

Brain-behavior associations have been examined separately. Some studies have found a significant correlation between atypical network organization and autistic traits (Glerean et al., 2016; Bolton et al., 2020), whereas others did not (Turner et al., 2025), highlighting variability across studies.

When compared with the resting-state literature, connectivity findings from naturalistic movie-viewing studies both converge with and extend prior findings about network organization in ASD. Resting-state studies have frequently reported reduced connectivity in long-range DMN connections linking medial PFC, posterior cingulate cortex (PCC), and inferior parietal gyrus (IPG), alongside atypical interactions between the DMN and the executive control network (Kennedy and Courchesne, 2008; Assaf et al., 2010; Hull et al., 2017). At the same time, several studies suggest increased connectivity between sensory cortices and subcortical regions, with findings often varying by age and analytical method (Hull et al., 2017).

Naturalistic connectivity studies replicate some of these large-scale patterns, including reduced connectivity within the DMN and altered sensory network involvement (Bolton et al., 2018, 2020; Ou et al., 2022). However, they additionally demonstrate that connectivity between temporoparietal and medial prefrontal regions fluctuates as a function of social and emotional content during movie viewing (e.g., Bolton et al., 2020). By linking connectivity changes to specific stimulus segments, these studies clarify when divergence emerges rather than only whether networks differ on average.

This temporal perspective is particularly relevant in autism, where difficulties often arise in integrating social information across extended periods of time rather than in responding to isolated events (Hedger and Chakrabarti, 2021; Vogel et al., 2022). Although methods in this field vary widely, their results consistently suggest large-scale network reorganization in ASD. This reorganization appears most evident during socially meaningful moments and highlights the importance of using naturalistic stimuli to understand brain function in real-world contexts.

### 3.4 Conventional analyses reveal further group differences

Although most naturalistic neuroimaging studies in autism have utilized model-free approaches such as ISC and ISFC, some investigations have applied conventional model-based analyses to continuous stimuli (e.g., Pantelis et al., 2015; Ross et al., 2024). These approaches typically use general linear modeling (GLM) or event-segmented contrasts to isolate responses to specific scenes or features embedded within naturalistic stimuli. While they may not fully exploit the dynamic nature of naturalistic stimuli, they allow more direct comparison with traditional task-based findings and can clarify whether group differences observed under controlled conditions extend to more ecologically valid contexts.

Rosenblau et al. (2016) used a GLM to examine amygdala responses to movie clips depicting mentalizing scenarios. Autistic individuals showed reduced amygdala activation relative to non-autistic individuals. Similarly, Scherf et al. (2010) examined neural responses to dynamic social stimuli and reported altered activation patterns in fusiform and temporal regions in adolescents with ASD. Pantelis et al. (2015) found that adults with ASD exhibited reduced activity in the TPJ and superior temporal sulcus (STS) when watching a TV show. Kotila et al. (2020) further identified group differences in socially relevant cortical regions during movie viewing, indicating that activation differences persist even when stimuli are not segmented into discrete experimental trials. In contrast, Ross et al. (2024) demonstrated that individuals with ASD exhibit increased frontal activation in response to continuous narrative speech not only when presented in an audiovisual format, but also when presented in a visual-only or audio-only format. This consistency suggests that increased engagement of higher-order processes during narrative comprehension may be consistent across modalities.

Taken together, these findings largely converge with prior conventional studies showing reduced amygdala or fusiform activation in response to facial stimuli and emotional expressions (Pierce and Redcay, 2008; Corbett et al., 2009; Sato et al., 2019; Wei et al., 2025; Kim et al., 2024). This convergence may be partly due to the analytic approach, i.e., by modeling discrete events, these studies constrain the naturalistic stimuli and therefore reproduce patterns observed under more controlled paradigms.

Converging evidence also comes from non-fMRI modalities. In a MEG study employing short movie clips, Matsuzaki et al. (2022) found that children and adolescents with ASD exhibited higher-amplitude responses to short movie clips in occipital and temporal cortices compared to non-autistic peers. Importantly, the magnitude of these neural differences correlated with individual differences in sensory sensitivity. These findings are consistent with prior sensory-processing paradigms (Marco et al., 2011; Patil and Kaple, 2023) and suggest that atypical sensory responses are evident during passive viewing of dynamic stimuli. Time-frequency analysis further revealed atypical oscillatory responses in autistic individuals across multiple frequency bands, consistent with the broader evidence of altered neural oscillatory dynamics in ASD (Matsuzaki et al., 2022).

Importantly, these model-based analyses complement rather than duplicate model-free findings. Whereas ISC and ISFC analyses quantify temporal alignment and network organization, conventional analyses identify regional differences in activation magnitude.

## 4 Discussion

This systematic review synthesized neuroimaging evidence from twenty studies employing naturalistic stimuli in ASD, revealing that autistic individuals respond to dynamic social stimuli in a more idiosyncratic manner than non-autistic individuals. This divergence is most pronounced in brain regions supporting social cognition, theory of mind, and self-referential processing. The most consistently reported finding is reduced ISC in frontoparietal and default mode regions, observed across developmental stages and across different experimental designs. Importantly, this reduction in between-subject synchrony appears to coexist with relatively preserved within-subject reliability, suggesting that autistic neural responses are not noisy but differently organized.

Functional connectivity analyses extend this picture by showing that network communication, particularly within the DMN and between temporoparietal and prefrontal regions, is less systematic in ASD during movie viewing, and that this reorganization fluctuates with the social and emotional content of the stimulus. Conventional analyses further confirm group differences in activation magnitude in regions such as the amygdala, fusiform cortex, TPJ, and STS. Together, these findings suggest that paradigms employing naturalistic stimuli are sensitive to neural differences in ASD that span social cognition, sensory processing, and large-scale network organization, and that some of these differences are linked to individual autistic characteristics and social comprehension scores. In several studies, group differences were not uniformly distributed across the stimulus but intensified during segments involving social interaction, mental state inference, or emotionally salient events. This is consistent with everyday observations that social inference demands vary considerably across context.

These findings both converge with and extend the broader ASD neuroimaging literature. The regions most consistently showing reduced ISC, including the anterior cingulate, medial prefrontal, insular, temporoparietal, and fusiform cortices, overlap substantially with areas implicated by task-based and resting-state meta-analyses (Di Martino et al., 2014; Patriquin et al., 2016; Chen et al., 2024). Similarly, reduced connectivity within the DMN and altered sensory network involvement replicate patterns reported in resting-state studies (Kennedy and Courchesne, 2008; Assaf et al., 2010; Hull et al., 2017). What studies with naturalistic stimuli add is temporal resolution at the level of narrative. Rather than reporting only whether networks differ on average, they reveal when divergence emerges and whether it is sustained or context-dependent. This emphasis on temporal dynamics is particularly relevant for autism, where difficulties often arise in integrating social information over extended periods rather than in responding to isolated events (Hedger and Chakrabarti, 2021; Vogel et al., 2022).

Despite this convergence, several methodological limitations constrain interpretation of the current literature. First, only a minority of studies have incorporated time-resolved stimulus annotations or explicit feature modeling (Pantelis et al., 2015; Bolton et al., 2020; Mangnus et al., 2025). Without explicit modeling of stimulus features, it is difficult to determine whether observed group differences reflect altered emotional processing, social evaluation, attentional engagement, narrative comprehension, or low-level perceptual processing. Future studies would benefit from integrating behavioral ratings obtained from independent cohorts (Pantelis et al., 2015) or from the same participants after scanning (Nummenmaa et al., 2012) to more precisely link neural dynamics to specific stimulus properties. Concurrent eye tracking would further strengthen this approach by providing a continuous, objective measure of visual attention deployment during movie viewing, which is particularly relevant in ASD, where gaze patterns during social scenes often differ from those of non-autistic individuals (Klin et al., 2002; Keles et al., 2022) and may partly explain neural divergence (Ramot et al., 2020). More systematic use of computational feature extraction, such as speech envelope tracking (Hausfeld et al., 2024) or automated face presence tracking (Ntoumanis et al., 2023), could further reduce interpretational ambiguity by quantifying stimulus properties directly rather than inferring them *post hoc*.

Second, within the ASD naturalistic neuroimaging literature identified by this review, fMRI is the dominant modality. Nineteen of the twenty included studies used fMRI and only one used MEG. Although fMRI offers excellent spatial precision, it provides limited insight into the temporal dynamics of neural responses to continuous stimuli. It is worth noting that EEG has been used alongside naturalistic video stimuli outside ASD research to measure attentional engagement during movie viewing (Dmochowski et al., 2012) and to characterize neural responses to videos in children with motor dysfunction (Ntoumanis et al., 2022). Similarly, time-resolved ISC analysis in MEG has revealed atypical neural processing of natural speech in other neurodevelopmental conditions (Thiede et al., 2020), illustrating the potential of such methods to dissociate early perceptual from later integrative processing stages. Electrophysiological modalities also permit analysis within distinct frequency bands, each associated with different cognitive states (Buzsáki and Draguhn, 2004; Giraud and Poeppel, 2012), and oscillatory dynamics in ASD can be examined through time-frequency analysis (Styliadis et al., 2021). Expanding the use of naturalistic stimuli to MEG and EEG could clarify whether reduced synchronization and altered connectivity arise at early sensory stages, during higher-order integration, or both.

Third, ISC and connectivity analyses are typically performed over the entire ASD sample, which may mask heterogeneity within the spectrum. A promising direction would be to stratify participants based on autistic characteristics (e.g., as in Lyons et al., 2020) or to apply machine learning clustering to identify subgroups based on neural synchronization profiles and then link these to behavioral phenotypes. Such approaches could move the field beyond binary group contrasts toward a more individualized understanding of neural mechanisms across the autism spectrum.

It is also important to acknowledge that movies, although more ecologically valid than static laboratory stimuli, are highly structured, professionally edited artifacts that guide attention and emotion through cinematography, music, and narrative design (Smith, 2012; Grall and Finn, 2022). Neural differences observed during movie viewing may therefore reflect divergence in how individuals respond to structured narrative cues rather than to spontaneous social engagement. Nevertheless, movies currently represent one of the most effective compromises between ecological complexity and experimental control (Nastase et al., 2019; Jääskeläinen et al., 2021), allowing exact stimulus replication across participants and precise annotation of content. These advantages are difficult to achieve in unconstrained real-world settings. A related concern is that ISC and ISFC patterns may be movie-specific, raising questions about the generalizability of findings obtained with a single stimulus. Future studies would benefit from including multiple movies to assess the replicability of neural synchronization patterns across different narrative contexts (Ye et al., 2023). Future work may also directly compare resting-state and movie-viewing paradigms within the same participants with ASD to clarify whether externally constrained conditions attenuate or amplify group differences (Finn et al., 2017; Finn and Bandettini, 2021).

The review process also has limitations that should be acknowledged. The review was not prospectively registered and no protocol was prepared. Due to the narrative nature of the synthesis and the heterogeneity of methods and outcome measures across studies, no formal assessment of reporting bias or certainty of evidence was conducted. These limitations should be considered when interpreting the findings.

Overall, naturalistic neuroimaging has shifted the focus of autism research from identifying isolated event-related differences to examining how neural responses evolve during shared social experiences. The current evidence suggests that autistic individuals process dynamic social stimuli in a consistently idiosyncratic manner, and that this divergence is most pronounced during moments of high social and affective demand. These findings point to neural systems that are particularly sensitive to naturalistic social information. These systems may be especially relevant for understanding real-world social difficulties in autism. Looking forward, longitudinal studies could examine whether changes in intersubject synchronization track developmental trajectories or responses to behavioral or neurofeedback-based interventions, and expanded use of temporally resolved neuroimaging modalities could clarify the mechanisms underlying the group differences reported here. Translating these findings into tools that support assessment and treatment in children with ASD remains an important goal.

## Data Availability

The original contributions presented in the study are included in the article/Supplementary material, further inquiries can be directed to the corresponding author.

## References

[ref1] American Psychiatric Association (2013). Diagnostic and Statistical Manual of mental Disorders. 5th Edn. Arlington, Virginia: American Psychiatric Publishing.

[ref2] Assaf M. Jagannathan K. Calhoun V. D. Miller L. Stevens M. C. Sahl R. . (2010). Abnormal functional connectivity of default mode sub-networks in autism spectrum disorder patients. NeuroImage 53, 247–256. doi: 10.1016/j.neuroimage.2010.05.067, 20621638 PMC3058935

[ref3] Benkarim O. Paquola C. Park B. Y. Hong S. J. Royer J. Vos de Wael R. . (2021). Connectivity alterations in autism reflect functional idiosyncrasy. Commun. Biol. 4:1078. doi: 10.1038/s42003-021-02572-6, 34526654 PMC8443598

[ref4] Bolton T. A. W. Jochaut D. Giraud A. L. Van De Ville D. (2018). Brain dynamics in ASD during movie-watching show idiosyncratic functional integration and segregation. Hum. Brain Mapp. 39, 2391–2404. doi: 10.1002/hbm.24009, 29504186 PMC5969252

[ref5] Bolton T. A. W. Freitas L. G. A. Jochaut D. Giraud A. L. Van De Ville D. (2020). Neural responses in autism during movie watching: inter-individual response variability co-varies with symptomatology. NeuroImage 216:116571. doi: 10.1016/j.neuroimage.2020.116571, 31987996

[ref6] Bressler S. L. Menon V. (2010). Large-scale brain networks in cognition: emerging methods and principles. Trends Cogn. Sci. 14, 277–290. doi: 10.1016/j.tics.2010.04.004, 20493761

[ref7] Buzsáki G. Draguhn A. (2004). Neuronal oscillations in cortical networks. Science 304, 1926–1929. doi: 10.1126/science.1099745 15218136

[ref8] Byrge L. Dubois J. Tyszka J. M. Adolphs R. Kennedy D. P. (2015). Idiosyncratic brain activation patterns are associated with poor social comprehension in autism. J. Neurosci. 35, 5837–5850. doi: 10.1523/JNEUROSCI.5182-14.2015, 25855192 PMC4388936

[ref9] Camacho M. C. Balser D. H. Furtado E. J. Rogers C. E. Schwarzlose R. F. Sylvester C. M. . (2024). Higher intersubject variability in neural response to narrative social stimuli among youth with higher social anxiety. J. Am. Acad. Child Adolesc. Psychiatry 63, 549–560. doi: 10.1016/j.jaac.2023.08.020, 38070872 PMC12035772

[ref10] Chen Y. Xi Z. Saunders R. Simmons D. Totsika V. Mandy W. (2024). A systematic review and meta-analysis of the relationship between sensory processing differences and internalising/externalising problems in autism. Clin. Psychol. Rev. 114:102516. doi: 10.1016/j.cpr.2024.102516, 39515075

[ref11] Chevallier C. Kohls G. Troiani V. Brodkin E. S. Schultz R. T. (2012). The social motivation theory of autism. Trends Cogn. Sci. 16, 231–239. doi: 10.1016/j.tics.2012.02.007, 22425667 PMC3329932

[ref12] Cooper N. Bassett D. S. Falk E. B. (2017). Coherent activity between brain regions that code for value is linked to the malleability of human behavior. Sci. Rep. 7:43250. doi: 10.1038/srep43250, 28240271 PMC5327429

[ref13] Cooper K. Russell A. Calley S. Chen H. Kramer J. Verplanken B. (2022). Cognitive processes in autism: repetitive thinking in autistic versus non-autistic adults. Autism 26, 849–858. doi: 10.1177/13623613211034380, 34291680 PMC9014768

[ref14] Corbett B. A. Carmean V. Ravizza S. Wendelken C. Henry M. L. Carter C. . (2009). A functional and structural study of emotion and face processing in children with autism. Psychiatry Res. 173, 196–205. doi: 10.1016/j.pscychresns.2008.08.005, 19665877 PMC2748131

[ref15] Czekóová K. Mareček R. Staněk R. Hartley C. Kessler K. Hlavatá P. . (2025). Altered patterns of dynamic functional connectivity underpin reduced expressions of social-emotional reciprocity in autistic adults. Autism Res 18, 725–740. doi: 10.1002/aur.70010, 39994920 PMC12015814

[ref16] Di Martino A. Yan C. G. Li Q. Denio E. Castellanos F. X. Alaerts K. . (2014). The autism brain imaging data exchange: towards a large-scale evaluation of the intrinsic brain architecture in autism. Mol. Psychiatry 19, 659–667. doi: 10.1038/mp.2013.78, 23774715 PMC4162310

[ref1101] Dmochowski J. P. Sajda P. Dias J. Parra L. C. (2012). Correlated components of ongoing EEG point to emotionally laden attention - a possible marker of engagement? Front. Hum. Neurosci, 6:112. doi: 10.3389/fnhum.2012.00112 22623915 PMC3353265

[ref17] Esfahlani F. Z. Byrge L. Tanner J. Sporns O. Kennedy D. P. Betzel R. F. (2022). Edge-centric analysis of time-varying functional brain networks with applications in autism spectrum disorder. NeuroImage 263:119591. doi: 10.1016/j.neuroimage.2022.119591, 36031181 PMC12403185

[ref18] Finn E. S. Scheinost D. Finn D. M. Shen X. Papademetris X. Constable R. T. (2017). Can brain state be manipulated to emphasize individual differences in functional connectivity? NeuroImage 160, 140–151. doi: 10.1016/j.neuroimage.2017.03.064, 28373122 PMC8808247

[ref19] Finn E. S. Glerean E. Khojandi A. Y. Nielson D. Molfese P. J. Handwerker D. A. . (2020). Idiosynchrony: from shared responses to individual differences during naturalistic neuroimaging. NeuroImage 215:116828. doi: 10.1016/j.neuroimage.2020.116828, 32276065 PMC7298885

[ref20] Finn E. S. Bandettini P. A. (2021). Movie-watching outperforms rest for functional connectivity-based prediction of behavior. NeuroImage 235:117963. doi: 10.1016/j.neuroimage.2021.117963, 33813007 PMC8204673

[ref21] Giraud A. L. Poeppel D. (2012). Cortical oscillations and speech processing: emerging computational principles and operations. Nat. Neurosci. 15, 511–517. doi: 10.1038/nn.3063, 22426255 PMC4461038

[ref22] Glerean E. Pan R. K. Salmi J. Kujala R. Lahnakoski J. M. Roine U. . (2016). Reorganization of functionally connected brain subnetworks in high-functioning autism. Hum. Brain Mapp. 37, 1066–1079. doi: 10.1002/hbm.23084, 26686668 PMC6867362

[ref23] Grall C. Finn E. S. (2022). Leveraging the power of media to drive cognition: a media-informed approach to naturalistic neuroscience. Soc. Cogn. Affect. Neurosci. 17, 598–608. doi: 10.1093/scan/nsac019, 35257180 PMC9164202

[ref24] Hasson U. Nir Y. Levy I. Fuhrmann G. Malach R. (2004). Intersubject synchronization of cortical activity during natural vision. Science 303, 1634–1640. doi: 10.1126/science.1089506, 15016991

[ref25] Hasson U. Avidan G. Gelbard H. Vallines I. Harel M. Minshew N. . (2009). Shared and idiosyncratic cortical activation patterns in autism revealed under continuous real-life viewing conditions. Autism Res. 2, 220–231. doi: 10.1002/aur.89, 19708061 PMC2775929

[ref26] Hasson U. Honey C. J. (2012). Future trends in neuroimaging: neural processes as expressed within real-life contexts. NeuroImage 62, 1272–1278. doi: 10.1016/j.neuroimage.2012.02.004, 22348879 PMC3360990

[ref27] Hausfeld L. Hamers I. M. H. Formisano E. (2024). fMRI speech tracking in primary and non-primary auditory cortex while listening to noisy scenes. Commun. Biol. 7:1217. doi: 10.1038/s42003-024-06913-z, 39349723 PMC11442455

[ref28] Hedger N. Chakrabarti B. (2021). Autistic differences in the temporal dynamics of social attention. Autism 25, 1615–1626. doi: 10.1177/1362361321998573, 33706553 PMC8323332

[ref29] Hernandez L. M. Rudie J. D. Green S. A. Bookheimer S. Dapretto M. (2015). Neural signatures of autism spectrum disorders: insights into brain network dynamics. Neuropsychopharmacology 40, 171–189. doi: 10.1038/npp.2014.172, 25011468 PMC4262896

[ref30] Hull J. V. Dokovna L. B. Jacokes Z. J. Torgerson C. M. Irimia A. Van Horn J. D. (2017). Resting-state functional connectivity in autism spectrum disorders: a review. Front. Psych. 7:205. doi: 10.3389/fpsyt.2016.00205, 28101064 PMC5209637

[ref31] Jääskeläinen I. P. Sams M. Glerean E. Ahveninen J. (2021). Movies and narratives as naturalistic stimuli in neuroimaging. NeuroImage 224:117445. doi: 10.1016/j.neuroimage.2020.117445, 33059053 PMC7805386

[ref32] Jääskeläinen I. P. Ahveninen J. Klucharev V. Shestakova A. N. Levy J. (2022). Behavioral experience-sampling methods in neuroimaging studies with movie and narrative stimuli. Front. Hum. Neurosci. 16:813684. doi: 10.3389/fnhum.2022.813684, 35153706 PMC8828971

[ref33] Keles U. Kliemann D. Byrge L. Saarimäki H. Paul L. K. Kennedy D. P. . (2022). Atypical gaze patterns in autistic adults are heterogeneous across but reliable within individuals. Mol. Autism. 13:39. doi: 10.1186/s13229-022-00517-2, 36153629 PMC9508778

[ref34] Kennedy D. P. Courchesne E. (2008). The intrinsic functional organization of the brain is altered in autism. NeuroImage 39, 1877–1885. doi: 10.1016/j.neuroimage.2007.10.052, 18083565

[ref35] Kim S. Y. Udhnani M. Lecavalier L. (2024). Heterogeneity in autism spectrum disorder explained by social-communicative and restricted repetitive behavior balance subgroups. Res. Autism Spectr. Disord. 114:102387. doi: 10.1016/j.rasd.2024.102387

[ref36] Klin A. Jones W. Schultz R. Volkmar F. Cohen D. (2002). Visual fixation patterns during viewing of naturalistic social situations as predictors of social competence in individuals with autism. Arch. Gen. Psychiatry 59, 809–816. doi: 10.1001/archpsyc.59.9.809, 12215080

[ref37] Kotila A. Hyvärinen A. Mäkinen L. Leinonen E. Hurtig T. Ebeling H. . (2020). Processing of pragmatic communication in ASD: a video-based brain imaging study. Sci. Rep. 10:21739. doi: 10.1038/s41598-020-78874-2, 33303942 PMC7729953

[ref38] Liberati A. Altman D. G. Tetzlaff J. Mulrow C. Gøtzsche P. C. Ioannidis J. P. . (2009). The PRISMA statement for reporting systematic reviews and meta-analyses of studies that evaluate health care interventions: explanation and elaboration. PLoS Med. 6:e1000100. doi: 10.1371/journal.pmed.1000100, 19621070 PMC2707010

[ref39] Lin F. Albantakis L. Noppari T. Santavirta S. Brandi M. L. Sun L. . (2026). Reduced inter-subject functional connectivity during movies in autism: replicability across cross-national fMRI datasets. Mol. Autism. 17:11. doi: 10.1186/s13229-026-00707-2, 41703633 PMC12922298

[ref41] Lord C. Brugha T. S. Charman T. Cusack J. Dumas G. Frazier T. . (2020). Autism spectrum disorder. Nat. Rev. Dis. Primers 6:5. doi: 10.1038/s41572-019-0138-4, 31949163 PMC8900942

[ref42] Lyons K. M. Stevenson R. A. Owen A. M. Stojanoski B. (2020). Examining the relationship between measures of autistic traits and neural synchrony during movies in children with and without autism. NeuroImage Clin. 28:102477. doi: 10.1016/j.nicl.2020.102477, 33395970 PMC7680702

[ref43] Mangnus M. Koch S. B. J. Cai K. Greidanus Romaneli M. Hagoort P. Bašnáková J. . (2025). Preserved spontaneous mentalizing amid reduced intersubject variability in autism during a movie narrative. Biol. Psychiatry Cogn. Neurosci. Neuroimaging 10, 1057–1066. doi: 10.1016/j.bpsc.2024.10.007, 39490786

[ref44] Marco E. J. Hinkley L. B. Hill S. S. Nagarajan S. S. (2011). Sensory processing in autism: a review of neurophysiologic findings. Pediatr. Res. 69, 48R–54R. doi: 10.1203/PDR.0b013e3182130c54, 21289533 PMC3086654

[ref45] Martynenko I. Koldewyn K. Downing P. E. (2025). Social brain responses to natural scene images of social interactions. Soc. Cogn. Affect. Neurosci. 20:nsaf057. doi: 10.1093/scan/nsaf057, 40465416 PMC12342172

[ref46] Matsuzaki J. Kagitani-Shimono K. Aoki S. Hanaie R. Kato Y. Nakanishi M. . (2022). Abnormal cortical responses elicited by audiovisual movies in patients with autism spectrum disorder with atypical sensory behavior: a magnetoencephalographic study. Brain Dev. 44, 81–94. doi: 10.1016/j.braindev.2021.08.007, 34563417

[ref47] McMahon E. Isik L. (2023). Seeing social interactions. Trends Cogn. Sci. 27, 1165–1179. doi: 10.1016/j.tics.2023.09.001, 37805385 PMC10841760

[ref48] McPartland J. C. Lerner M. D. Bhat A. Clarkson T. Jack A. Koohsari S. . (2021). Looking back at the next 40 years of ASD neuroscience research. J. Autism Dev. Disord. 51, 4333–4353. doi: 10.1007/s10803-021-05095-5, 34043128 PMC8542594

[ref49] Meer J. N. V. Breakspear M. Chang L. J. Sonkusare S. Cocchi L. (2020). Movie viewing elicits rich and reliable brain state dynamics. Nat. Commun. 11:5004. doi: 10.1038/s41467-020-18717-w, 33020473 PMC7536385

[ref50] Moola S. Munn Z. Tufanaru C. Aromataris E. Sears K. Sfetcu R. . (2020). “Chapter 7: systematic reviews of etiology and risk,” in JBI Manual for Evidence Synthesis, eds. Aromataris E. Munn Z. (Adelaide, Australia).

[ref51] Nastase S. A. Gazzola V. Hasson U. Keysers C. (2019). Measuring shared responses across subjects using intersubject correlation. Soc. Cogn. Affect. Neurosci. 14, 667–685. doi: 10.1093/scan/nsz037, 31099394 PMC6688448

[ref52] Nomi J. S. Uddin L. Q. (2015). Developmental changes in large-scale network connectivity in autism. NeuroImage Clin. 7, 732–741. doi: 10.1016/j.nicl.2015.02.024, 25844325 PMC4375789

[ref53] Northoff G. Heinzel A. de Greck M. Bermpohl F. Dobrowolny H. Panksepp J. (2006). Self-referential processing in our brain – a meta-analysis of imaging studies on the self. NeuroImage 31, 440–457. doi: 10.1016/j.neuroimage.2005.12.002, 16466680

[ref54] Ntoumanis I. Agranovich O. Shestakova A. N. Blagovechtchenski E. Koriakina M. Kadieva D. . (2022). Altered cerebral processing of videos in children with motor dysfunction suggests broad embodiment of perceptual cognitive functions. J. Pers. Med. 12:1841. doi: 10.3390/jpm12111841, 36579567 PMC9697218

[ref55] Ntoumanis I. Shestakova A. Koriakina M. Kadieva D. Kopytin G. Jääskeläinen I. P. (2023). Developmental differences in the perception of naturalistic human movements. Front. Hum. Neurosci. 16:1046277. doi: 10.3389/fnhum.2022.1046277, 36704095 PMC9872020

[ref56] Ntoumanis I. Sheronova J. Davydova A. Dolgaleva M. Jääskeläinen I. P. Kosonogov V. . (2024). Deciphering the neural responses to a naturalistic persuasive message. Proc. Natl. Acad. Sci. USA 121:e2401317121. doi: 10.1073/pnas.2401317121, 39413130 PMC11513929

[ref57] Ntoumanis I. Townsend M. Cooper C. M. Papadelis C. (2026). Rapid engagement of salience and prefrontal systems during emotional processing in children: an MEG study. NeuroImage 328:121806. doi: 10.1016/j.neuroimage.2026.121806, 41690336

[ref58] Nummenmaa L. Glerean E. Viinikainen M. Jääskeläinen I. P. Hari R. Sams M. (2012). Emotions promote social interaction by synchronizing brain activity across individuals. Proc. Natl. Acad. Sci. USA 109, 9599–9604. doi: 10.1073/pnas.1206095109, 22623534 PMC3386135

[ref59] Ou W. Zeng W. Gao W. He J. Meng Y. Fang X. . (2022). Movie events detecting reveals inter-subject synchrony difference of functional brain activity in autism spectrum disorder. Front. Comput. Neurosci. 16:877204. doi: 10.3389/fncom.2022.877204, 35591883 PMC9110681

[ref60] Pantelis P. C. Byrge L. Tyszka J. M. Adolphs R. Kennedy D. P. (2015). A specific hypoactivation of right temporo-parietal junction/posterior superior temporal sulcus in response to socially awkward situations in autism. Soc. Cogn. Affect. Neurosci. 10, 1348–1356. doi: 10.1093/scan/nsv021, 25698698 PMC4590532

[ref61] Patil O. Kaple M. (2023). Sensory processing differences in individuals with autism spectrum disorder: a narrative review of underlying mechanisms and sensory-based interventions. Cureus 15:e48020. doi: 10.7759/cureus.48020, 38034138 PMC10687592

[ref62] Patriquin M. A. DeRamus T. Libero L. E. Laird A. Kana R. K. (2016). Neuroanatomical and neurofunctional markers of social cognition in autism spectrum disorder. Hum. Brain Mapp. 37, 3957–3978. doi: 10.1002/hbm.23288, 27329401 PMC5053857

[ref63] Pelphrey K. A. Shultz S. Hudac C. M. Vander Wyk B. C. (2011). Research review: constraining heterogeneity: the social brain and its development in autism spectrum disorder. J. Child Psychol. Psychiatry 52, 631–644. doi: 10.1111/j.1469-7610.2010.02349.x, 21244421 PMC3096715

[ref64] Philip R. C. Dauvermann M. R. Whalley H. C. Baynham K. Lawrie S. M. Stanfield A. C. (2012). A systematic review and meta-analysis of the fMRI investigation of autism spectrum disorders. Neurosci. Biobehav. Rev. 36, 901–942. doi: 10.1016/j.neubiorev.2011.10.008, 22101112

[ref65] Pierce K. Redcay E. (2008). Fusiform function in children with an autism spectrum disorder is a matter of "who". Biol. Psychiatry 64, 552–560. doi: 10.1016/j.biopsych.2008.05.013, 18621359 PMC2673799

[ref66] Ramot M. Walsh C. Reimann G. E. Martin A. (2020). Distinct neural mechanisms of social orienting and mentalizing revealed by independent measures of neural and eye movement typicality. Commun. Biol. 3:48. doi: 10.1038/s42003-020-0771-1, 31996763 PMC6989525

[ref67] Redcay E. Schilbach L. (2019). Using second-person neuroscience to elucidate the mechanisms of social interaction. Nat. Rev. Neurosci. 20, 495–505. doi: 10.1038/s41583-019-0179-4, 31138910 PMC6997943

[ref68] Rolls E. T. Huang C. C. Lin C. P. Feng J. Joliot M. (2020). Automated anatomical labelling atlas 3. NeuroImage 206:116189. doi: 10.1016/j.neuroimage.2019.116189, 31521825

[ref69] Rosenblau G. Kliemann D. Lemme B. Walter H. Heekeren H. R. Dziobek I. (2016). The role of the amygdala in naturalistic mentalising in typical development and in autism spectrum disorder. Br. J. Psychiatry 208, 556–564. doi: 10.1192/bjp.bp.114.159269, 26585095

[ref70] Ross L. A. Molholm S. Butler J. S. Del Bene V. A. Brima T. Foxe J. J. (2024). Neural correlates of audiovisual narrative speech perception in children and adults on the autism spectrum: a functional magnetic resonance imaging study. Autism Res. 17, 280–310. doi: 10.1002/aur.3104, 38334251

[ref71] Saarimäki H. (2021). Naturalistic stimuli in affective neuroimaging: a review. Front. Hum. Neurosci. 15:675068. doi: 10.3389/fnhum.2021.675068, 34220474 PMC8245682

[ref72] Salmi J. Roine U. Glerean E. Lahnakoski J. Nieminen-von Wendt T. Tani P. . (2013). The brains of high functioning autistic individuals do not synchronize with those of others. NeuroImage Clin. 3, 489–497. doi: 10.1016/j.nicl.2013.10.011, 24273731 PMC3830058

[ref74] Sato W. Kochiyama T. Uono S. Yoshimura S. Kubota Y. Sawada R. . (2019). Atypical amygdala-neocortex interaction during dynamic facial expression processing in autism spectrum disorder. Front. Hum. Neurosci. 13:351. doi: 10.3389/fnhum.2019.00351, 31680906 PMC6813184

[ref75] Scherf K. S. Luna B. Minshew N. Behrmann M. (2010). Location, location, location: alterations in the functional topography of face- but not object- or place-related cortex in adolescents with autism. Front. Hum. Neurosci. 4:26. doi: 10.3389/fnhum.2010.00026, 20631857 PMC2904054

[ref76] Schurz M. Radua J. Aichhorn M. Richlan F. Perner J. (2014). Fractionating theory of mind: a meta-analysis of functional brain imaging studies. Neurosci. Biobehav. Rev. 42, 9–34. doi: 10.1016/j.neubiorev.2014.01.009, 24486722

[ref77] Simony E. Honey C. J. Chen J. Lositsky O. Yeshurun Y. Wiesel A. . (2016). Dynamic reconfiguration of the default mode network during narrative comprehension. Nat. Commun. 7:12141. doi: 10.1038/ncomms12141, 27424918 PMC4960303

[ref78] Smith T. J. (2012). The attentional theory of cinematic continuity. PRO 6, 1–27. doi: 10.3167/proj.2012.060102

[ref79] Sonkusare S. Breakspear M. Guo C. (2019). Naturalistic stimuli in neuroscience: critically acclaimed. Trends Cogn. Sci. 23, 699–714. doi: 10.1016/j.tics.2019.05.004, 31257145

[ref80] Styliadis C. Leung R. Özcan S. Moulton E. A. Pang E. Taylor M. J. . (2021). Atypical spatiotemporal activation of cerebellar lobules during emotional face processing in adolescents with autism. Hum. Brain Mapp. 42, 2099–2114. doi: 10.1002/hbm.25349, 33528852 PMC8046060

[ref81] Tang S. Sun N. Floris D. L. Zhang X. Di Martino A. Yeo B. T. T. (2020). Reconciling dimensional and categorical models of autism heterogeneity: a brain connectomics and behavioral study. Biol. Psychiatry 87, 1071–1082. doi: 10.1016/j.biopsych.2019.11.009, 31955916

[ref82] Thiede A. Glerean E. Kujala T. Parkkonen L. (2020). Atypical MEG inter-subject correlation during listening to continuous natural speech in dyslexia. NeuroImage 216:116799. doi: 10.1016/j.neuroimage.2020.116799, 32294536

[ref83] Tian J. Gao X. Yang L. (2022). Repetitive restricted behaviors in autism spectrum disorder: from mechanism to development of therapeutics. Front. Neurosci. 16:780407. doi: 10.3389/fnins.2022.780407, 35310097 PMC8924045

[ref84] Turner J. M. Byrge L. Richardson H. Galdi P. Kennedy D. P. Kliemann D. (2025). Social inference brain networks in autistic adults during movie-viewing: functional specialization and heterogeneity. Mol. Autism. 16:42. doi: 10.1186/s13229-025-00669-x, 40846984 PMC12372314

[ref85] Uddin L. Q. Supekar K. Menon V. (2013). Reconceptualizing functional brain connectivity in autism from a developmental perspective. Front. Hum. Neurosci. 7:458. doi: 10.3389/fnhum.2013.00458, 23966925 PMC3735986

[ref86] Vanderwal T. Kelly C. Eilbott J. Mayes L. C. Castellanos F. X. (2015). Inscapes: a movie paradigm to improve compliance in functional magnetic resonance imaging. NeuroImage 122, 222–232. doi: 10.1016/j.neuroimage.2015.07.069, 26241683 PMC4618190

[ref87] Vandewouw M. M. Dunkley B. T. Lerch J. P. Anagnostou E. Taylor M. J. (2021). Characterizing inscapes and resting-state in MEG: effects in typical and atypical development. NeuroImage 225:117524. doi: 10.1016/j.neuroimage.2020.117524, 33147510

[ref88] Vogel D. H. V. Jording M. Esser C. Conrad A. Weiss P. H. Vogeley K. (2022). Temporal binding of social events less pronounced in individuals with autism spectrum disorder. Sci. Rep. 12:14853. doi: 10.1038/s41598-022-19309-y, 36050371 PMC9437002

[ref89] Volkmar F. R. (2011). Understanding the social brain in autism. Dev. Psychobiol. 53, 428–434. doi: 10.1002/dev.20556, 21678390

[ref90] Wei L. Zhou M. Hu P. Jia S. Zhong S. (2025). Abnormal brain activation in autism spectrum disorder during negative emotion processing: a meta-analysis of functional neuroimaging studies. J. Psychiatr. Res. 185, 1–10. doi: 10.1016/j.jpsychires.2025.03.032, 40138749

[ref91] Ye M. Liu J. Guan Y. Ma H. Tian L. (2023). Are inter-subject functional correlations consistent across different movies? Brain Imaging Behav. 17, 44–53. doi: 10.1007/s11682-022-00740-8, 36418674

[ref94] Zimmer L. Richardson H. Pletti C. Paulus M. Schuwerk T. (2025). Predictive responses in the theory of mind network: a comparison of autistic and non-autistic adults. Cortex 187, 159–171. doi: 10.1016/j.cortex.2025.04.006, 40373360

